# Early Teacher–Child Relationships Promote Self-Regulation Development in Prekindergarten

**DOI:** 10.3390/ijerph19148802

**Published:** 2022-07-20

**Authors:** Kathleen Moritz Rudasill, Ibrahim Acar, Yaoying Xu

**Affiliations:** 1 School of Education, Virginia Commonwealth University, Richmond, VA 23284, USA; yxu2@vcu.edu; 2 Department of Psychology, Faculty of Social Sciences, Özyeğin University, Istanbul 34794, Turkey; ibrahim.acar@ozyegin.edu.tr

**Keywords:** teacher–child relationship, self-regulation, effortful control, prekindergarten

## Abstract

Children’s experiences during the prekindergarten period are critical for shaping their emerging self-regulation skills. The purpose of this study was to examine the contribution of teacher–child relationship quality to children’s performance on a self-regulation task at the end of prekindergarten. Teachers rated the conflict, closeness, and dependency in their relationships with 104 children in the fall of prekindergarten, and children’s self-regulation was independently measured with a visual attention task in the spring of prekindergarten. In addition, teachers and parents rated children’s temperamental self-regulation (i.e., effortful control). Results indicate that greater teacher–child dependency predicted children’s longer time on the visual attention task, and greater teacher–child closeness predicted children’s lower accuracy on the visual attention task. In addition, children who were rated as more self-regulated by parents were more accurate on the visual attention task. The implications of the results are discussed.

## 1. Introduction

When children start kindergarten, they are expected to be ready for the demands of the classroom environment, such as sharing, listening, following directions, and sitting still. Yet, at the start of kindergarten, nearly 50% of kindergarten teachers report that half or more of their students lack key skills, such as following directions (Rimm-Kaufman et al., 2000) [1]. These key skills are undergirded by self-regulation which develops rapidly from ages 3 to 7 years, spanning the ages during which children attend prekindergarten and kindergarten (Montroy et al., 2016; Rothbart and Bates, 2006) [2,3]. Thus, children’s experiences during this period are critical for shaping their emerging self-regulation skills. Children who attend prekindergarten programs have an advantage when it comes to developing self-regulation skills that are especially helpful in the school environment, such as paying attention to teacher instructions and taking turns (Blair and Diamond, 2008; Shapiro, 2021; Williford et al., 2013) [4,5,6].

However, children’s prekindergarten experiences vary widely (Shapiro, 2021) [5] and research suggests that children with high quality relationships with their prekindergarten teachers may be better prepared academically and socially for formal schooling than children with poorer quality relationships (Chiu and Chi, 2014; Pianta, 1999; Rau et al., 2015; Rimm-Kaufman and Pianta, 2000) [7,8,9,10]. The purpose of this study was to examine the contribution of teacher–child relationship quality to children’s performance in a self-regulation task at the end of prekindergarten. We hypothesized that positive teacher–child relationships during prekindergarten cultivate children’s comfort in the learning environment, thus encouraging them to explore and experiment in the classroom, which maximizes their self-regulation development. Children with negative teacher–child relationships, on the other hand, may feel less comfortable or insecure, and have suboptimal self-regulation development.

### 1.1. Self-Regulation

Self-regulation is critical for children’s successful navigation of the school environment (Blair, 2002) [11]. As outlined by McClelland and Cameron (2012) [12], there are three key components of self-regulation that work together to facilitate children’s optimal behavior in the classroom. These are: attentional flexibility—the ability to focus and sustain attention as needed; working memory—remembering key information during tasks and activities, such as teacher directions; and inhibitory control—the ability to refrain from inappropriate behavior and enact appropriate behavior, such as asking for an object instead of snatching the object. Broadly, self-regulation and the associated skills—attention, working memory, and inhibitory control—are often referred to as executive function (Miyake et al., 2000) [13] which has been implicated in children’s abilities to control behavior, attention, thought, and emotion (e.g., Zelazo et al., 2010) [14]. Likewise, attention and inhibitory control are core constructs of effortful control (Rothbart and Bates, 2006) [3], defined as temperamentally based self-regulation that is conceptualized as modulating children’s more reactive temperament traits (e.g., motor activity, anger) for a given environment. Executive function is typically measured with a direct assessment of a child’s task performance, whereas effortful control is typically measured through parent, teacher, or caregiver reports. Children’s self-regulation in preschool has been empirically linked to their academic performance concurrently and longitudinally (Blair, 2016; McClelland et al., 2007) [15,16]. Research shows that children who enter kindergarten lacking in self-regulation rarely catch up; instead, the achievement gap tends to widen over time (e.g., Bulotsky-Shearer et al., 2016) [17], particularly for children who are from culturally and/or linguistically diverse backgrounds.

### 1.2. Teacher–Child Relationships

The teacher–child relationship in prekindergarten serves a critical function for children’s development, as this is typically the first important relationship a child has in the school environment. The teacher–child relationship sets the tone for a child’s perceptions of the classroom and school (e.g., positive or negative, safe or unsafe, fun or boring). In early childhood, the quality of this relationship is usually assessed via teacher reports, and conceptualized as having three dimensions: closeness, conflict, and dependency. Positive teacher–child relationships are conceptualized as those with high levels of closeness and low levels of conflict and dependency, whereas negative relationships are those low in closeness and high in conflict and/or dependency. Closeness is characterized by warmth and mutual adoration between teacher and child. Conflict is marked by frustration or distrust, and dependency is noted when the teacher feels that the child is overly needy, asking unnecessary questions or persistently seeking the teacher’s presence or assistance (Pianta, 2001) [18]. Importantly, dependency is a far less often measured dimension of the teacher–child relationship, and has not consistently been linked to negative outcomes for children. Indeed, the valence of dependency seems to be at least partially a function of culture; in interdependent cultures (e.g., Portugal), dependency is positively correlated with closeness and viewed as protective (Ferreira et al., 2021) [19], but in independent cultures (e.g., USA) dependency is viewed as problematic (Tsigilis et al., 2018) [20]. Since the current study took place in the US, we conceptualized dependency as a component of negative teacher–child relationships.

### 1.3. Teacher–Child Relationships and Self-Regulation

Teacher–child relationships are foundational to children’s growing self-regulation skills in school (Williford et al., 2013) [6]. According to Williford et al., “when children interact with teachers who establish positive emotional bonds with and meet the behavioral and regulatory needs of their students, this creates an environment that should be especially supportive for children’s ability to self-regulate” (pp. 165–166). When these relationships are positive, teachers provide a secure base from which children can explore the classroom environment, and feel comfortable and safe as they grow in their abilities and knowledge (Pianta, 1999) [8]. When teacher–child relationships are negative, however, children feel unstable or uncertain about the learning environment; they may be reluctant to try new things, or miss out on opportunities for skill development (Pianta, 1999) [8].

The extant literature indicates that teacher–child relationship quality and self-regulation in early childhood are, indeed, connected both concurrently and longitudinally (see Vandenbroucke et al., 2017 for a meta-analysis) [21]. Regarding concurrent associations, Abenavoli and Greenberg (2014) [22] found that kindergarteners’ conflict with teachers was negatively related to their self-regulation (i.e., inhibitory control, the ability to inhibit inappropriate behaviors), and closeness was positively related to self-regulation (i.e., inhibitory control and attention shifting, the ability to shift attention to stimuli as required by the environment). In addition, closeness was linked to better working memory performance, another facet of self-regulation.

Similarly, Commodari (2013) [23] examined associations between children’s secure attachment to preschool teachers, based on trained observers’ ratings on an attachment Q-sort, and their concurrent self-regulation (i.e., attention) skills. Children’s accuracy on an attention task was better when they were observed to be more securely attached to their preschool teachers. Specifically, more securely attached children had more accuracy in terms of auditory recognition, visual recognition, visual spatial recognition, and divided attention tasks. Less securely attached children took longer than their more securely attached peers to complete auditory recognition, multiple search letter, and multiple search symbol tasks.

Studies of longitudinal links between the quality of teacher–child relationships and children’s self-regulation revealed similar trends. Cadima et al. (2016) [24] found that teacher-reported teacher–child closeness predicted growth in children’s self-regulation across the prekindergarten year. Using a child report of teacher–child relationship quality in a study across kindergarten and first grade, de Wilde et al. (2016) [25] found not only that teacher–child conflict predicted poorer working memory, but also that better working memory predicted lower child-perceived conflict with teachers and higher warmth (i.e., closeness). Ferreira et al. (2021) [19] examined the longitudinal linkages between children’s relationships with teachers (based on teacher report) and effortful control across two years of preschool in Portugal. Teacher–child dependency in year 1 was predictive of effortful control (based on teacher report) in year 2, such that greater dependency predicted higher effortful control.

Recent work illustrates the broader connections between teacher–child interactions and children’s self-regulation in the prekindergarten period. For example, in a study of teacher–child interaction quality in prekindergarten, Hatfield et al. (2022) [26] revealed that higher quality classroom interactions predicted better self-regulation among children. Elsewhere, Acar and colleagues (2022) [27] found that teacher–child closeness moderated the association between children’s hot self-regulation (i.e., self-regulation tasks tied to a reward) and learning behaviors (attention/persistence, attitude toward learning, competence/motivation). That is, children with higher “hot” self-regulation and more closeness in the teacher–child relationship tended to display more learning behaviors. Taken together, the extant literature supports linkages between teacher–child relationship quality and children’s self-regulation in prekindergarten.

### 1.4. The Present Study

Collectively, research suggests that early teacher–child relationships are associated with children’s self-regulation skill development. However, studies of self-regulation and teacher–child relationship quality are limited in several ways. First, many studies are cross-sectional, thus limiting the capacity to draw temporally based causal conclusions (e.g., Abenavoli and Greenberg, 2014; Cadima et al., 2016; Commodari, 2013) [22,23,24]. Second, some studies are longitudinal but situate self-regulation as the hypothesized predictor of teacher–child relationship quality (e.g., Spilt and Hughes, 2015) [28]. Such examinations are illuminating, but do not interrogate the extent to which teacher–child relationship quality, a classroom characteristic that may be addressed with teacher training and professional development, may contribute to a child’s development of self-regulation. Third, many studies take place after, rather than during, prekindergarten when significant self-regulation development occurs (e.g., Berry, 2012; de Wilde et al., 2016) [25,29]. Finally, only one of the existing studies included the dependency dimension of the teacher–child relationship (Ferreira et al., 2021) [19]. Dependency is far less examined in the teacher–child relationship literature than closeness and conflict, and research suggests that it is not a uniformly negative relationship dimension for all children (Rudasill, 2021) [30]. Thus, it is important to develop a better understanding of how teacher–child dependency may be related to children’s self-regulation during this critical, prekindergarten period.

In the current study, self-regulation was measured with the Nepsy visual attention task which is an assessment of a child’s ability to attend to visual stimuli (attention), retain information during a task (working memory), and inhibit inappropriate behaviors in favor of required behavior (inhibitory control). Ability in this task matures earlier than some other self-regulation skills, with six-year-olds performing similarly to adults (Visu-Petra et al., 2007) [31]. Thus, at the age of four years, we expect this skill to be developing rapidly. The Nepsy visual attention score is based on the number of correct items identified (accuracy) and the time taken to finish the assessment (time). These scores are inversely correlated (*r* = 0.51, Visu-Petra et al., 2007) [31]. We postulated that children’s relationships with their teachers would help them build confidence and a sense of safety in exploring the classroom for a foundation of self-regulation to be extended, thus providing more growth. We separated these scores because, given their inverse correlation, we were interested in their distinct associations with teacher-child relationship quality. Previous studies of children’s attention show that accuracy and time are differentially related to children’s relationships with teachers (e.g., Commodari, 2013) [23]. We also controlled for demographic variables (gender, family income). In addition, we were interested in the extent to which parent and teacher ratings of children’s effortful control in the fall of prekindergarten were good predictors of children’s performance on the attention task in spring of the prekindergarten year.

## 2. Method

### 2.1. Participants

We recruited 104 children (56 females, 48 males) from a small Midwestern city in the United States. Children were enrolled in 23 classrooms (22 female teachers, 1 male teacher; all White) from 9 preschools representing state-supported, private, and religiously affiliated programs. All children in the current study were developing typically as reported by parents. All prekindergarten children from these schools were invited to participate in the study; approximately 50% of parents provided their consent for their child to participate. Due to limited resources for data collection, a maximum of seven children from each class was included in the study; in classrooms where more than seven children had consent to participate, seven children were randomly selected. Between one and seven children per classroom participated (*M* = 4.5, *SD* = 1.6). Children’s ages ranged from 32 to 63 months (*M* = 50 months, *SD* = 6.22) at the beginning of the study. Parents reported children’s race/ethnicity which were as follows: White (*n* = 80; 76.9%), Latino (*n* = 5; 4.8%), Asian (*n* = 3; 2.9%), African American/Black (*n* = 2; 1.9%), and mixed race (*n* = 13; 12.5%). Annual family income was reported by parents using a scale from 1 = <$5000 to 11 = >$95,000 (M = 8, $65,000–75,000).

### 2.2. Child Temperament

Parents and teachers rated children’s effortful control on the following subscales of the Children’s Behavior Questionnaire (CBQ; Rothbart et al., 2001) [32]: Inhibitory Control and Attentional Focusing. The CBQ has a 7-point scale from 1 = *extremely untrue of your child* to 7 = *extremely true of your child.* Sample items and internal consistency estimates for each subscale are as follows: Inhibitory Control (“*Is usually able to resist temptation when told s/he is not supposed to do something*”, α = 0.75 for parent report, α = 0.89 for teacher report, 13 items); Attentional Focusing (“*When picking up toys or other jobs, usually keeps at the task until it’s done*” α = 0.70 for parent report, α = 0.81 for teacher report, nine items). Based on previous research, the temperament-based rating of self-regulation, effortful control, was formed from the aggregate of inhibitory control and attentional focusing (e.g., Rudasill and Rimm-Kaufman 2009) [33], resulting in a variable for parent-reported effortful control and a variable for teacher-reported effortful control. Parent and teacher ratings of temperament were kept separate because previous studies indicated that parent and teacher ratings tend to have low agreement (Rudasill et al., 2014; Valiente et al., 2012) [34,35].

### 2.3. Teacher–Child Relationship

We used the Student–Teacher Relationship Scale (STRS; Pianta, 2001) [18] to measure teachers’ perceptions of their relationships with children in their classrooms. The STRS is a 28-item questionnaire where teachers rate their relationships with individual children on a 5-point scale (1 = *definitely does not apply* to 5 = *definitely does apply*). The STRS contains the following three subscales: Conflict, Closeness, and Dependency. The Conflict subscale has 12 items (e.g., “*This child and I always seem to be struggling with each other*”. α = 0.87). The Closeness subscale has 11 items (e.g., “*I share an affectionate, warm relationship with this child*”. α = 0.80). The Dependency subscale has 5 items (e.g., “*This child reacts strongly to separation from me*”. α = 0.48). We deleted one item due to low internal consistency. This decision is based on previous confirmatory factor analysis with these data (Rudasill & Acar, 2019) [36] showing that removal of item 14 (“*This child asks for my help when he/she really does not need help*”) improved model fit and resulted in an improved internal consistency (α = 0.58), congruent to the internal consistency found in previous studies (e.g., Hamre and Pianta, 2001) [37].

### 2.4. Children’s Self-Regulation

Children’s self-regulation was measured using the visual attention subtest of the NEPSY (Korkman et al., 1998) [38], which requires children to visually peruse an array of pictures of common items (e.g., flowers, dogs, cats) and find the target items (i.e., cats) within 180 s or less. Children first identified cats, then completed a second array to identify bunnies. This test measures children’s attentional flexibility (paying attention to one visual stimulus, then to another), working memory (holding in mind a particular visual stimulus for identification during a task), and inhibitory control (refraining from identifying pictures of incorrect stimuli). The following two scores were calculated: accuracy, which is calculated based on the number of target pictures identified minus the incorrectly identified pictures; and time, which is calculated based on the time the child takes to find the pictures within 180 s. The scores were inversely correlated (*r* = −0.51). Internal consistency across test administrations (cats and bunnies) was high for each score (accuracy (*r* = −0.90), time (*r* = 0.76)).

### 2.5. Procedure

This study was approved by the University’s Institutional Review Board. In the fall of the prekindergarten year, teachers received the STRS to complete for each study child in their classrooms. In addition, teachers and parents received the CBQ to complete for each study child. Researchers collected these measures along with teacher and parent consent forms upon completion. In the spring of the prekindergarten year, children’s self-regulation was assessed with the NEPSY visual attention task as part of a battery of direct assessments. Researchers met with children one-on-one in the school setting to administer the assessments after first obtaining child assent to participate.

### 2.6. Data Analysis

Initially, we tested the normality assumptions of the predictor variables in terms of skewness and kurtosis by using the using ±2 as criteria (Gravette and Wallnau, 2014; Trochim and Donnelly, 2006) [39,40]. Our predictor variables in the current study were within the acceptable range for normality assumptions, so no transformation was needed. Table 1 shows the relevant details. Second, the percentage of missing values ranged from 1% to 9.6% across variables. We used the Little’s (1988) [41] MCAR (Missing Completely at Random) test to understand the mechanism of the missing data. The results of the applied missing value analysis (MVA) showed that missing values were completely random (MCAR), *χ^2^* = 31.199, *p* = 0.183. In addition, we used the Restricted Maximum Likelihood (RML) method to handle missing data, because it allowed us to use any available data points on examined variables (Larsen, 2011) [42]. Considering the nested structure of our data (children within classrooms), we employed the “type is complex’’ procedure in Mplus for our regression models to account for nesting in classrooms (Muthen and Muthen 2017) [43]. The complex procedure provides us with accurate standard errors for estimations reflecting non-independence (i.e., multiple children within classrooms) (Muthén and Satorra 1995) [44]. The between-class (ICC) intraclass correlation was 0.10 for visual attention time (design effect = 1.342) and 0.26 for visual attention accuracy (design effect = 1.839).

## 3. Results

### 3.1. Visual Attention Accuracy

We regressed children’s visual attention accuracy on teacher–child relationships (dependency, closeness, and conflict) by controlling for demographic variables (child sex, child age, and annual family income) and parent/teacher-rated children’s effortful control. Teacher–child closeness significantly predicted children’s visual attention accuracy (*β* = −0.322, *t* = −3.808 (*95% CI* = −0.470/−0.148) *p* < 0.01). Thus, with every increase of one standard deviation in teacher–child closeness, children’s visual attention accuracy decreased by 0.322 standard deviations. In addition, parent-reported effortful control significantly predicted children’s visual attention accuracy (*β* = 0.322, *t* = 3.368 (*95% CI* = 0.156/0.491) *p* < 0.01). With every increase of one standard deviation in parent-reported effortful control, children’s visual attention accuracy increased by 0.322 standard deviations. See Table 2 for the complete results.

### 3.2. Visual Attention Time

We regressed children’s visual attention time on teacher–child relationships (dependency, closeness, and conflict) by controlling for demographic variables (child sex, child age, and annual family income) and parent/teacher-rated children’s effortful control. Teacher–child dependency significantly predicted children’s visual attention time (*β* = 0.266, *t* = 2.10 (*95% CI* = 0.006/0.516) *p* < 0.01). That is, with every increase of one standard deviation in teacher–child dependency, children’s visual attention time increased by 0.266 standard deviations.

## 4. Discussion

Three main findings emerged in this longitudinal study of prekindergarten children’s self-regulation as predicted by the quality of their relationships with their teachers. First, regarding children’s time on the Nepsy subtest, higher ratings of teacher–child dependency predicted longer time. Second, in predicting children’s accuracy on the visual attention task, children rated as being closer with their teachers were less accurate on the task. Third, parent ratings of children’s effortful control predicted children’s higher accuracy on the task. That is, children rated as more self-regulated by parents performed with more accuracy on the visual attention task. Each of these findings are discussed below.

First, we found that children rated as having more dependency in their relationships with prekindergarten teachers had longer time scores on the visual attention task. That is, children viewed as higher in dependency were also slower to complete the visual attention task. Slower times are indicative of lower attention control (Espy and Bull, 2005; Visu-Petra et al., 2007) [31,45]; a child who is able to attend well can complete the task in less time than a child who has difficulty sustaining attention. Thus, our finding that prekindergarten children have higher time scores when they are rated by teachers as more dependent is consistent with the literature linking teacher-child dependency and children’s poorer academic outcomes, especially in Western countries (e.g., Rudasill, 2021) [30]. Indeed, the extant literature suggests a small, negative association between teacher–child dependency and children’s academic achievement, and a medium, positive association between dependency and children’s externalizing and internalizing behavior (Roorda et al., 2011) [46]. Children viewed as highly dependent demonstrate careful or cautious behaviors that are characterized by teachers as overly needy; such children tend to ask a lot of questions that seem unnecessary, spend more time near the teacher than other children or than *with* other children, and seek teacher reassurance (Pianta, 2001) [18]. It could be that children who display these behaviors with teachers are also more cautious and careful as they completed the attention task, thus resulting in slower times. 

Our next result was that children who were rated by teachers as having closer relationships were also less accurate on the visual attention task. Children’s accuracy score on the visual attention task is an assessment of their ability to correctly identify a target picture (within allotted time), with deductions for errors. We expected children with higher levels of closeness with teachers to be more, not less, accurate. Thus, our finding indicates that children with more teacher-child closeness were less self-regulated in that they demonstrated less attentional control on this task. This is incongruent with the literature connecting teacher–child closeness with children’s positive academic outcomes (e.g., Kincade et al., 2020) [47]. It is possible this finding is an artifact of the visual attention task and we would have results more aligned with the literature showing positive associations between teacher-child closeness and self-regulation (e.g., Vandenbrouke et al., 2017) [21] with a different assessment of self-regulation. Alternatively, it could be that we found this unexpected result because we were examining closeness as a predictor of self-regulation development. Although there is robust support self-regulation as a predictor of teacher-child relationship quality, including closeness (e.g., Rudasill and Rimm-Kaufman, 2009) [33], the reverse has been less explored. In addition, there may be some child characteristics that explain this effect. For instance, children who are lower in shyness (i.e., bolder) are also likely to develop close relationships with teachers in preschool (e.g., Rudasill et al., 2006) [48]; bolder children also tend to be more boisterous and show less self-regulated behavior in class (Rimm-Kaufman et al., 2002) [49]. Thus, child boldness could be a moderating factor to explain our finding. Clearly, this result warrants further investigation. 

Our final result, that parent ratings of children’s effortful control were positively related to children’s accuracy on the visual attention task, is congruent with previous research connecting effortful control with self-regulation (Acar et al., 2021; Liew, 2011) [50,51]. Indeed, there is conceptual overlap between effortful control and the broader construct of self-regulation (Liew, 2011) [51]. As with self-regulation, the literature strongly suggests that children’s effortful control contributes positively to their academic and social success in school (e.g., Rudasill et al., 2013; Valiente et al., 2010; Zhou et al., 2012) [52,53,54]. Interestingly, teacher ratings of children’s self-regulation were unrelated to children’s task accuracy. Other research has shown a lower correlation between teacher and parent-reported temperament (e.g., Rudasill et al., 2014) [48].

## 5. Strengths, Limitations, and Future Research

This study provides insight into the ways that children’s early relationships with teachers, particularly teacher–child dependency and closeness, contribute to their developing self-regulation. There are several key strengths to the study. First, we used a longitudinal design to examine links between children’s relationships with teachers and their developing self-regulation from the beginning to the end of prekindergarten. Second, we used standardized and well-validated measures (i.e., STRS, CBQ, NEPSY). Finally, we controlled for potential confounding variables in our analyses. However, there are several limitations that should be considered. First, the sample was relatively racially/ethnically homogenous and somewhat socioeconomically privileged. Future research should prioritize the study of these questions with more diverse groups of children and teachers, with specific attention to groups who are under-represented and minoritized in psychological and educational research, such as non-White samples. Second, although this study was longitudinal across the prekindergarten year, another limitation is that data were not available to follow the children’s pathways into kindergarten. A future study could examine the longitudinal outcomes of children from prekindergarten through the early grades to determine the potential downstream benefits of teacher–child relationship quality and self-regulation on children’s academic and social adjustment through kindergarten. Third, this study relies on one, visual test of self-regulation. It may be that different results would emerge with a different type of self-regulation assessment. This work could be extended by examining the association between children’s relationships with prekindergarten teachers and their performance on Head-Toes-Knees-Shoulders (McClelland et al., 2021) [55], which is a brief, direct assessment that captures general self-regulation in prekindergarten aged children.

## 6. Conclusions

This study of prekindergarten self-regulation development adds to the extant literature by revealing several points. First, in the early education years, dependency in the teacher–child relationship may be problematic for children’s self-regulation development. Second, closeness in this relationship may also be problematic for children’s self-regulation. Although closeness has been widely found to be positive for children’s school-related outcomes, our findings indicate that there may be some additional factors at play in understanding the linkages between teacher-child closeness and the development of self-regulation. Prekindergarten teacher-child relationships are potential points of intervention for promoting positive self-regulation development, as well as possible bellwethers of self-regulation difficulties.

## Figures and Tables

**Table 1:** Descriptive Statistics and Intercorrelations among Study Variables.

Variable	1	2	3	4	5	6	7	8	9	10
1. VA_Time	-									
2. VA_Accuracy	0.50 **	-								
3. P-EC	−0.28 **	0.40 **	-							
4. T-EC	−0.15	0.24 *	0.28 **	-						
5. Dependency	0.23 *	−0.11	−0.20 ^†^	−0.11	-					
6. Closeness	−0.02	−0.02	0.11	0.48 **	0.11	-				
7. Conflict	−0.06	−0.18	−0.20 ^†^	−0.40 **	0.33 **	−0.28 **	-			
8. Age	−0.08	0.25 *	0.12	0.22 *	0.09	0.20 ^†^	−0.03	-		
9. Income	0.05	0.13	0.17	0.19	0.09	0.08	0.03	−0.01	-	
10. Child Sex	−0.13	0.08	0.18	0.26 **	0.02	0.12	−0.02	0.03	−0.02	-
*Mean*	173.84	24.76	4.86	4.46	2.23	3.97	2.03	50.05	0	
*SD*	71.64	34.28	0.81	1.07	0.76	0.50	0.68	6.67	1	
*Minimum*	64	−109	2.09	1.44	1	2	1.25	31	−2.17	
*Maximum*	360	40	6.67	6.75	4	5	4.33	63	0.83	
*Skewness*	0.905	−3.08	−0.490	−0.407	0.295	−0.990	1.33	−0.16	−0.82	
*Kurtosis*	0.193	8.37	0.362	0.147	−0.849	2.18	1.60	−0.15	−0.93z	

Note. * *p* < 0.05, ** *p* < 0.01. ^†^ *p* = 0.05. VA = Visual Attention, P = Parent Report, T = Teacher Report, EC = Effortful Control, Child Sex (1 = F 0 = M).

**Table 2:** Final model parameters for effortful control and teacher–child relationships predicting children’s visual time and accuracy.

	Visual Attention Time			Visual Attention Accuracy		
	*Unstandardized Estimate (SE)*	*Standardized Estimate (SE)*	*95% CI*	*Unstandardized Estimate (SE)*	*Standardized Estimate (SE)*	*95% CI*
Intercept	363.098 (102.031)	5.010 (1.399)	2.201/7.722	−14.287 (43.993)	−0.404 (1.259)	−2.73/2.05
Child Sex	−12.845 (17.379)	−0.088 (0.124)	−0.342/0.137	0.064 (7.520)	0.001 (0.108)	−0.191/0.222
Family Income	11.449 (8.217)	0.156 (0.111)	−0.079/0.368	0.650 (3.901)	0.018 (0.104)	−0.171/0.234
Child Age	−0.558 (1.335)	−0.053 (0.125)	−0.285/0.191	1.389 (0.835) *	0.271 (0.122)	0.013/0.474
P_Effortful Control	−22.733 (14.423)	−0.258 (0.167)	−0.559/0.073	13.882 (5.693) **	0.322 (0.096)	0.156/0.491
T_Effortful Control	−11.164 (10.328)	−0.155 (0.140)	−0.435/0.106	5.943 (4.245)	0.169 (0.123)	−0.094/0.417
T-C Dependency	24.904 (11.960) **	0.266 (0.132)	0.006/0.516	−1.352 (4.079)	−0.030 (0.101)	−0.235/0.173
T-C Closeness	3.853 (25.184)	0.023 (0.149)	−0.294/0.304	−26.900 (10.340) **	−0.322 (0.085)	−0.470/−0.148
T-C Conflict	−25.432 (17.399)	−0.238 (0.151)	−0.534/0.026	−7.555 (5.911)	−0.145 (0.125)	−0.360/0.170
*R^2^*	0.216			0.296		
*AIC*	946.386			818.527		
*BIC*	970.575			842.715		

* *p* < 0.05, ** *p* < 0.01. P = Parent-report, T = Teacher report. T-C = Teacher–Child. Child Sex (1 = F 0 = M). CI = Confidence Interval (2000 bootstrap).
